# Solitary myofibroma of the mandible in an adult with magnetic resonance imaging and positron emission tomography findings: a case report

**DOI:** 10.1186/1477-7819-12-69

**Published:** 2014-03-28

**Authors:** Yoko Tanaka, Hiroyuki Yamada, Tomoyuki Saito, Kazutoshi Nakaoka, Kenichi Kumagai, Hisako Fujihara, Kenji Mishima, Yoshiki Hamada

**Affiliations:** 1 Department of Oral and Maxillofacial Surgery, Sendai Tokushukai Hospital, Sendai, Miyagi, Japan; 2 Department of Oral and Maxillofacial Surgery, Tsurumi University School of Dental Medicine, 2-1-3 Tsurumi, Tsurumi-ku, Yokohama, Japan; 3 Department of Oral and Maxillofacial Surgery, Nagano Matsushiro General Hospital, Matsushiro, Nagano, Japan; 4 Department of Oral Pathology and Diagnosis, School of Dentistry, Showa University, Hatanodai, Shinagawa-ku, Tokyo, Japan

**Keywords:** Myofibroma, Mandible, Adult, MRI, PET

## Abstract

Myofibroma is a benign tumor composed of myoid spindle cells. The prevalence of myofibroma in the oral cavity is very low, with the mandible being the most common site. This report describes an adult case of myofibroma that arose on the mandible and includes magnetic resonance imaging (MRI) and positron emission tomography (PET) findings. On the MRI T1-weighted images, the tumor appeared with signal iso-intensity and was highly and heterogeneously enhanced with contrast material. On the T2-weighted images, it appeared with increased signal intensity. ^18^ F-fluorodeoxyglucose (FDG)-PET imaging showed abnormal strong accumulation of FDG in the left mandibular region. The tumor was removed by marginal resection of the left mandible under general anesthesia. Histopathological findings revealed that the tumor stroma contained abundant thin-walled vessels. The postoperative course was uneventful, and we found no evidence of recurrence at the postoperative 34-month follow-up.

## Background

Myofibroma is a benign tumor composed of myoid spindle cells arranged around thin-walled blood vessels. Myofibromas are classified as either a solitary or multicentric type [1]. The solitary type is called myofibroma, whereas the multicentric type is known as myofibromatosis. The majority of myofibromas present in children less than 2 years of age [2]. Approximately half of myofibromas occur in the cutaneous and subcutaneous tissues of the head and neck region, followed by the trunk and the extremities. The other half occur in skeletal muscle or aponeuroses [1]. The prevalence of myofibroma in the oral cavity is very low, and the mandible is recognized as the most common site followed by the tongue and buccal mucosa [3]. Therefore, there are few reports of adult cases of oral myofibroma [4].

Some cases of oral myofibroma have been misdiagnosed as various benign and malignant tumors [5-7] because oral pathologists are infrequently exposed to soft tissue spindle cell neoplasms coupled with overlapping histologic patterns [5]. Although many reports dealing with oral myofibroma limited their focus to its pathological features [8,9], reports describing preoperative diagnostic imaging findings are few [10]. A preoperative image-based diagnosis using magnetic resonance imaging (MRI) and positron emission tomography (PET) would be helpful to determine whether the tumor is benign or malignant and to determine the treatment plan. In this paper, we report an adult case of myofibroma that arose on the mandible and emphasize the diagnostic value of preoperative MRI and PET findings.

## Case presentation

**Figure 1:**
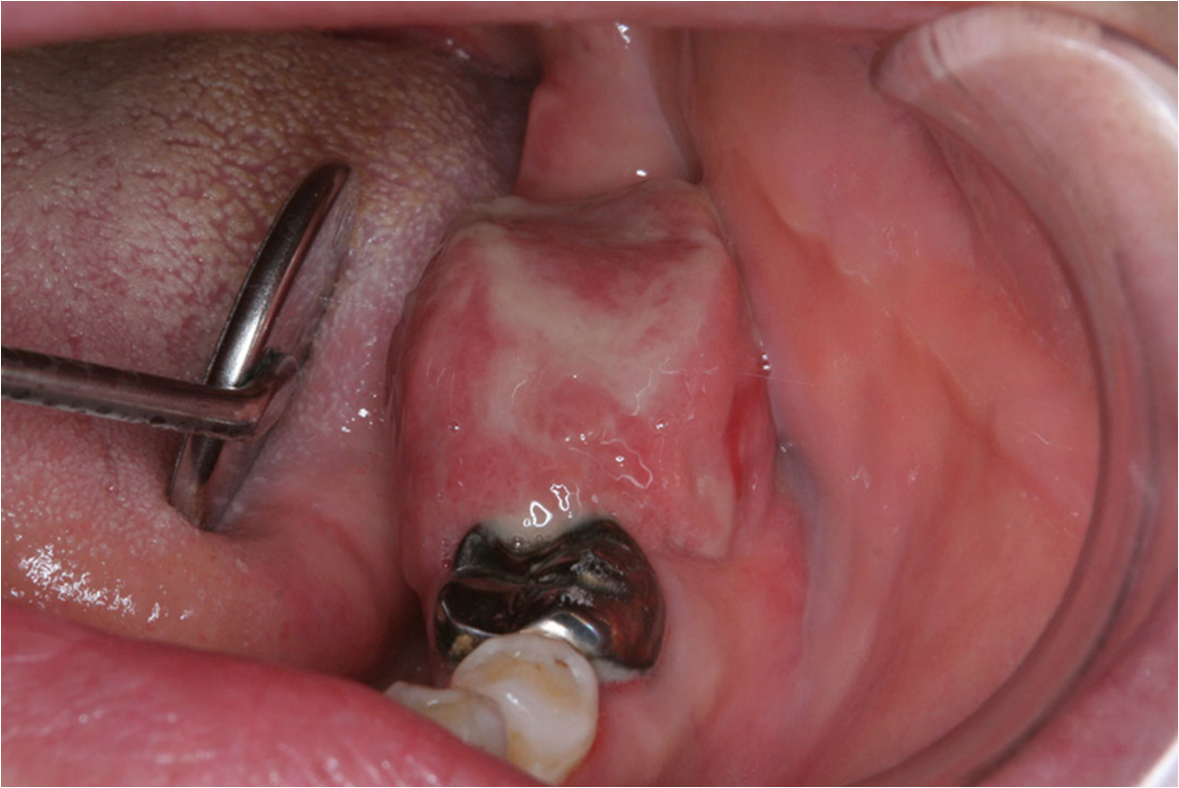
Photograph showing the mass on the mandible.

A 52-year-old woman was referred to the Department of Oral and Maxillofacial Surgery, Tsurumi University Dental Hospital (Yokohama, Japan) on 30 October 2010 for diagnosis and treatment of a mass on the left mandible. The patient had noticed the mass for 1 month. The mass grew rapidly from the extraction socket of the lower mandibular second molar. Past medical history revealed hypertension and hyperlipidemia, which were adequately controlled with medication. The patient’s family history was unremarkable. Physical examination revealed a rubbery sessile mass measuring 20 × 15 mm on the left mandible (Figure 1). The overlying oral mucosa looked absent. There was neither neuroparalysis nor cervical lymphadenopathy.

**Figure 2:**
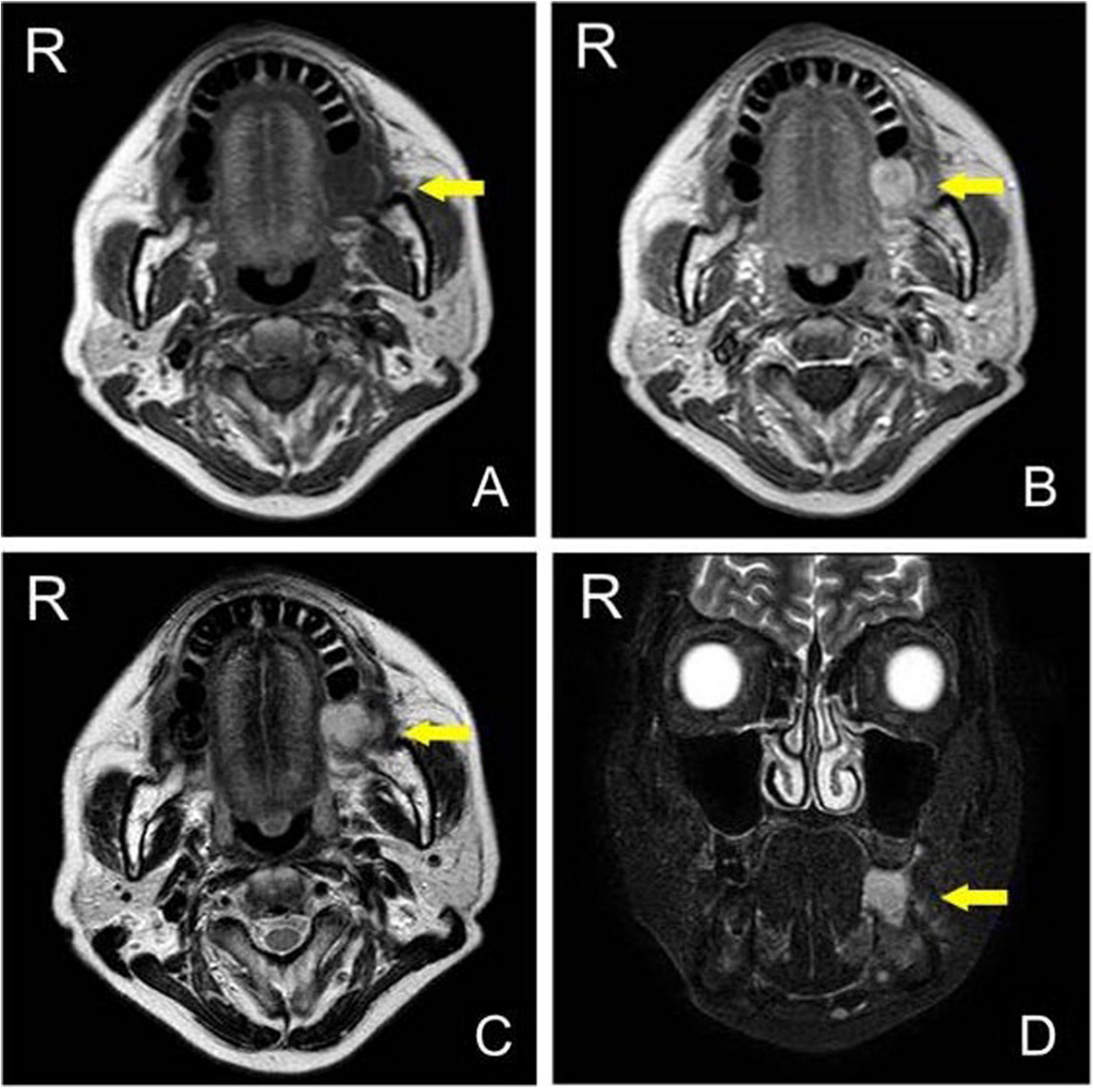
**Preoperative MRI images. (A)** T1-weighted axial image showing a mass of iso-intensity on the mandible (arrow). **(B)** Gadolinium-enhanced T1-weighted axial image showing a highly and heterogeneously enhanced mass (arrow). **(C)** T2-weighted axial image showing high signal intensity within the mass (arrow). **(D)** Short-tau inversion recovery images showing high signal intensity in the mass (arrow). MRI, magnetic resonance imaging.

**Figure 3:**
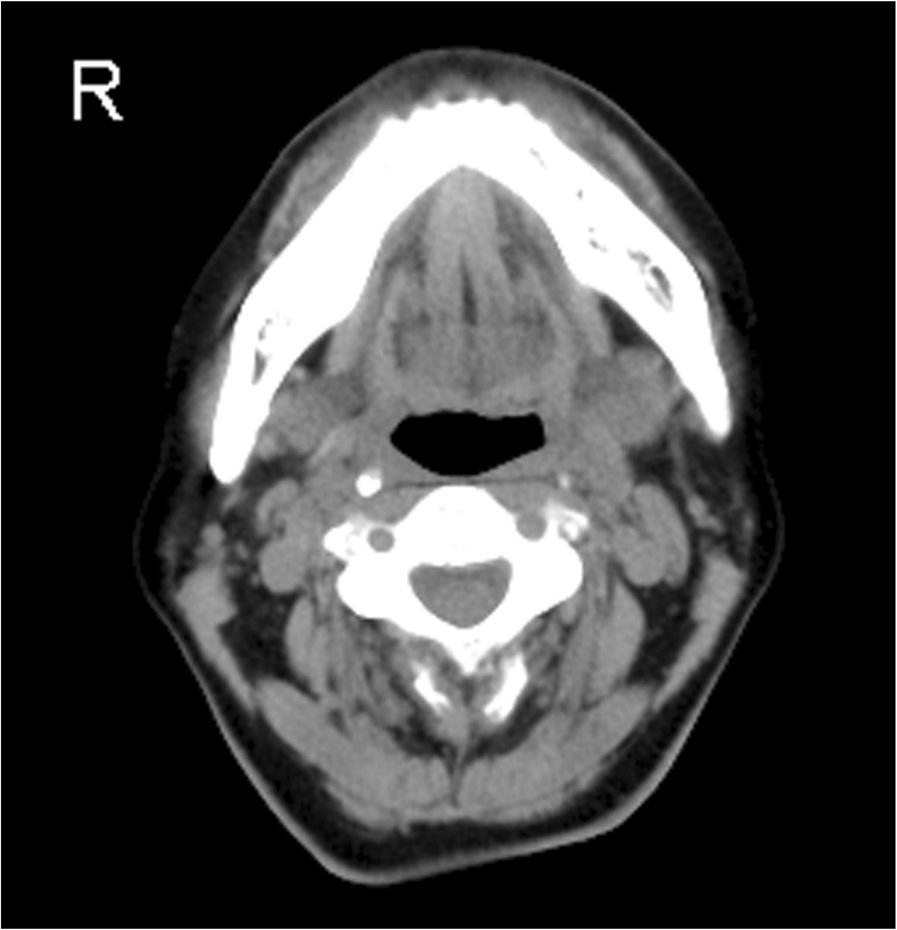
**CT image showing no bony changes of the mandible.** CT, computed tomography.

**Figure 4:**
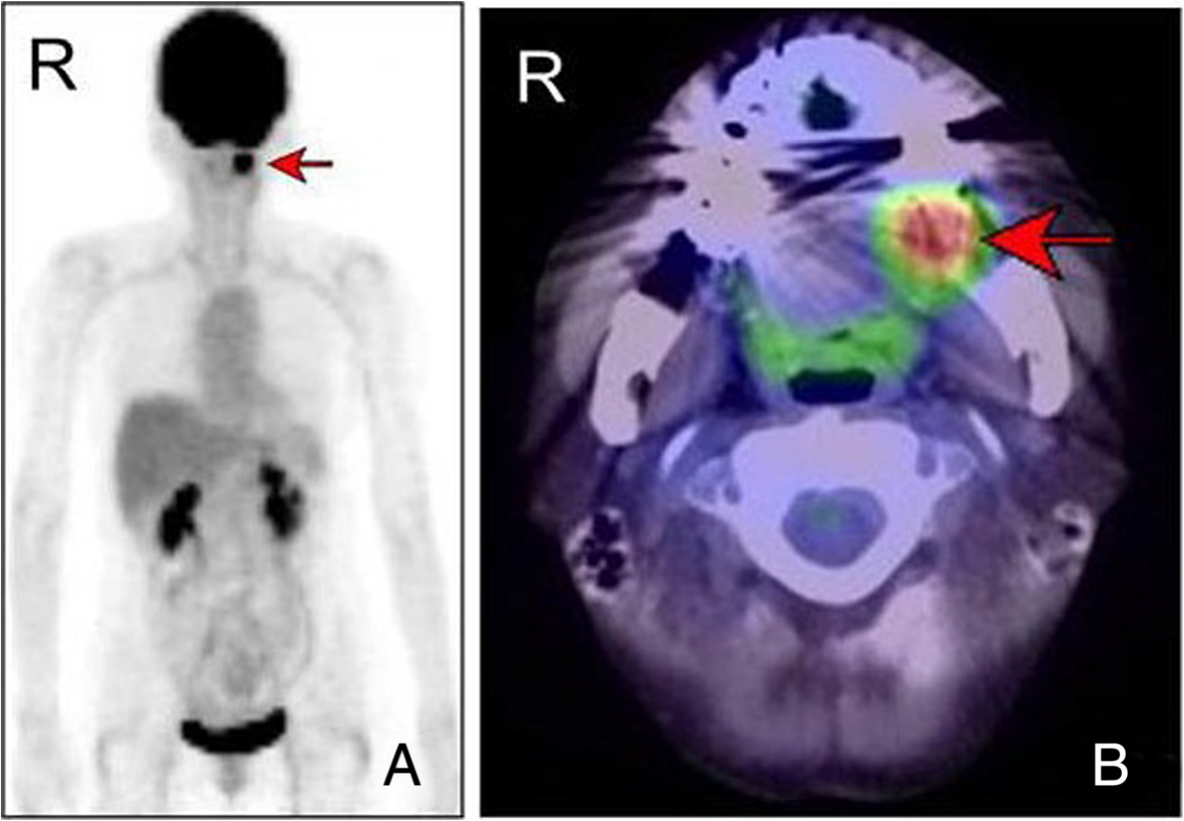
**FDG-PET and PET/CT images. (A)** FDG-PET coronal image of the mass. There is an abnormal accumulation of FDG in the left mandibular region (arrow). **(B)** PET/CT fusion image showing abnormal strong FDG accumulation corresponding to the mass on the left mandible (arrow). CT, computed tomography; FDG, ^18^ F-fluorodeoxyglucose; PET, positron emission tomography.

MRI examination was performed with a 1.5 Tesla system (Intera 1.5 T; Philips Healthcare, Eindhoven, The Netherlands). On the T1-weighted axial images, the mass appeared isointense and was highly and heterogeneously enhanced with gadolinium in the left mandibular region. On the T2-weighted axial images, it appeared with increased signal intensity. On short-tau inversion recovery coronal imaging, the mass also showed high signal intensity (Figure 2). The CT images (Brilliance CT 16; Philips Healthcare, Eindhoven, The Netherlands) showed no bony changes of the mandible (Figure 3). The PET study was performed with a dedicated PET scanner (ECAT ACCEL; Siemens Healthcare AG, Munich, Germany). Data were acquired after administration of 181.5 MBq ^18^ F-fluorodeoxyglucose (FDG), using the post-injection transmission acquisition method. The image showed abnormal strong accumulation of FDG in the left mandibular region with a maximum standard uptake value of 8.5. There were no abnormal accumulations of FDG in the cervical lymph nodes nor was there any uptake in the other organs (Figure 4A). The PET/CT fusion images showed that abnormal strong FDG accumulation corresponded to the mass on the left mandible (Figure 4B).

**Figure 5:**
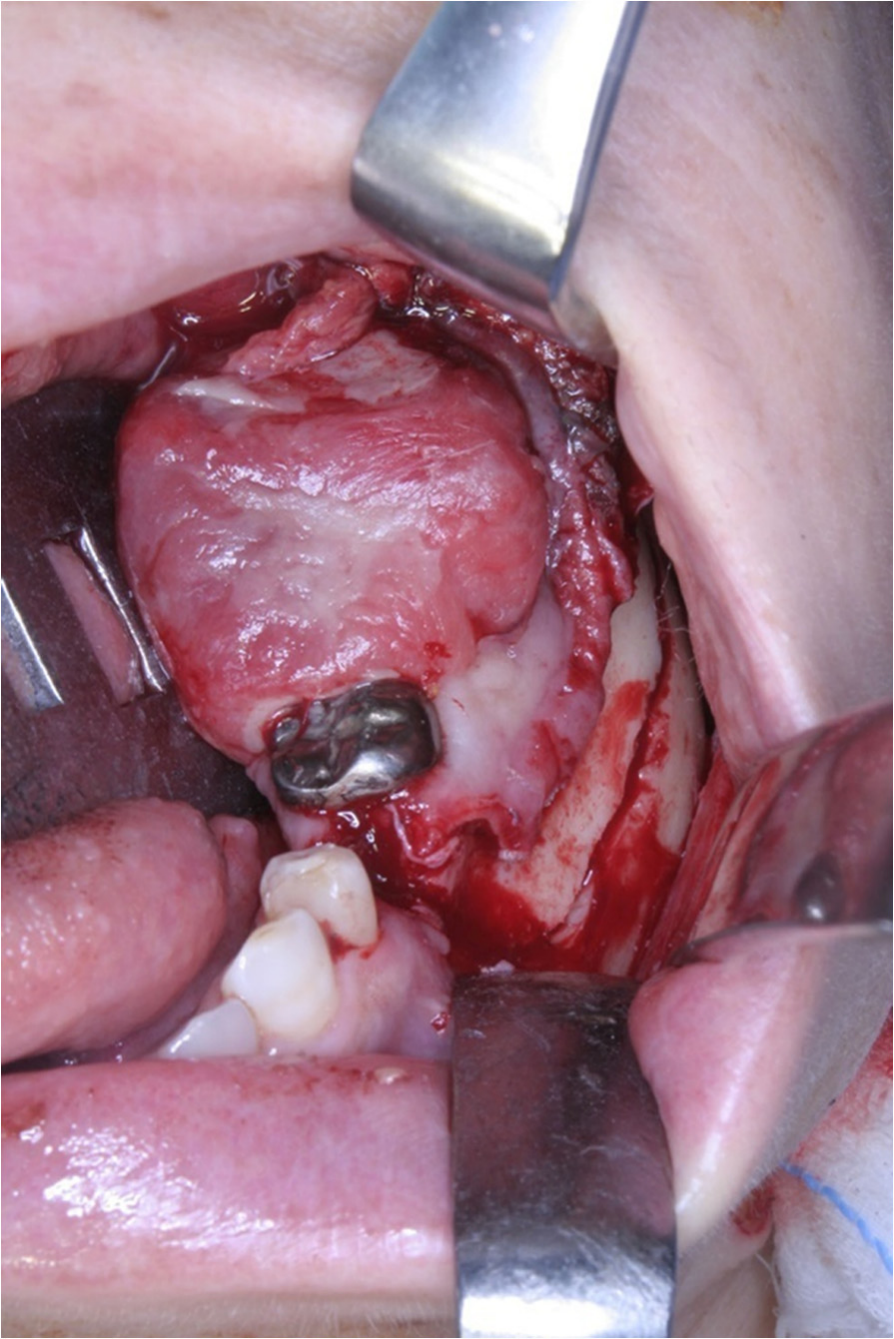
Photograph showing intraoperative view of the marginal resection of the left mandible.

In November 2010, the biopsy was performed under local anesthesia. The pathological diagnosis was myofibroblastic tumor, probably benign. However, the mass enlarged rapidly to 35 × 25 mm within 3 weeks after biopsy. According to the clinical findings, we could not completely deny the malignancy of the tumor. On 2 December 2010, it was removed by marginal resection of the left mandible under general anesthesia (Figure 5).

**Figure 6:**
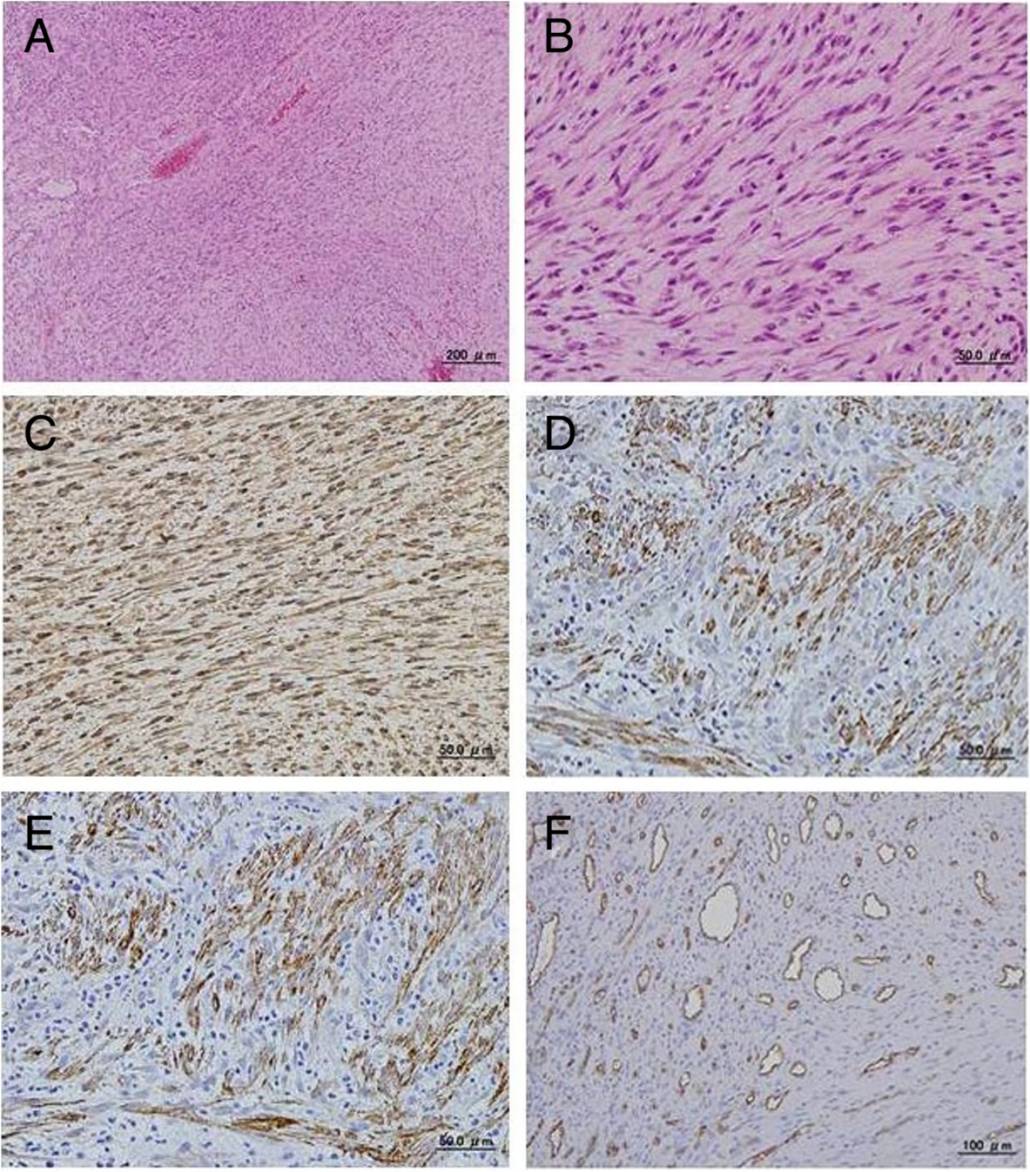
**Photomicrographs showing spindle-shaped cells, cytoplasmic immunoreactivity and vascular endothelial cells. (A)** Interlacing bundles of spindle-shaped cells (HE stain, original magnification × 100). **(B)** Spindle-shaped cells with tapered blunt-ended nuclei and pale-staining eosinophilic cytoplasm (HE stain, original magnification × 400). **(C)** Cytoplasmic positive immunoreactivity for vimentin (original magnification × 400). **(D)** Cytoplasmic positive immunoreactivity for HHF-35 (original magnification × 400). **(E)** Cytoplasmic positive immunoreactivity for α-SMA (original magnification × 400). **(F)** Vascular endothelial cells with cytoplasmic positive immunoreactivity for CD34 (original magnification × 200). HE, hematoxylin and eosin.

Pathological examination of hematoxylin and eosin (HE)-stained sections revealed that the tumor was composed of interlacing bundles of spindle-shaped cells. The tumor cells had tapered blunt-ended nuclei with pale-staining eosinophilic cytoplasm. The spindle-shaped cells did not show either cellular atypism or polymegethism. Mitoses were sometimes observed. The stroma contained abundant thin-walled vessels (Figure 6A,B). Immunohistochemical staining was performed using an avidin-biotin method. Immunoreactivity for vimentin (mouse anti-vimentin monoclonal antibody, clone V9; DakoCytomation, Glostrup, Denmark), muscle actin (mouse anti-human muscle actin monoclonal antibody, clone HHF35; DakoCytomation), and α-smooth muscle actin (mouse anti-human alpha smooth muscle actin monoclonal antibody, clone 1A4; Daco, Carpinteria, CA, USA) were positive in the tumor cells. Conversely, S-100 protein (rabbit anti-human S-100 polyclonal antibody, clone MOC32; Nichirei, Tokyo, Japan), desmin (mouse anti-human desmin monoclonal antibody, clone D33; Daco, Kyoto, Japan), and CD34 (mouse anti-human CD34 monoclonal antibody, clone NU-4A1; Nichirei, Tokyo, Japan) were negative. Thin-walled vessels were positive for CD34 (Figure 6C,D,E). These findings were consistent with the diagnosis of myofibroma.

The postoperative course was uneventful, and we found no evidence of recurrence at the postoperative 34-month follow-up.

## Discussion

There are few reports of adult patients with myofibroma of the mandible [4]. Clinical differences between adults and pediatric patients with myofibroma have suggested that pediatric patients are likely to demonstrate bone involvement [7,11] and that lesions in adults almost invariably presented as the solitary form, with exceptional cases of the multicentric form [6]. However, no specific differences in histologic features between adult and pediatric patients have been reported [6,11]. The clinical features of our solitary case without bone destruction are in accordance with those of adult oral myofibroma.

In the present case, the preoperative diagnosis based on the biopsy specimen findings was a myofibroblastic tumor, probably benign. However, the tumor grew rapidly in a few weeks. In the oral and maxillofacial region, approximately one-fourth of cases of myofibroma exhibit rapid enlargement, and some lesions show accelerated growth after incisional biopsy [11]. Spindle cell tumors are difficult to diagnose with a small piece of biopsy specimen alone [5,7]. Therefore, the final pathological diagnosis must rely on a specimen from the entire surgically removed lesion. Consequently, we performed marginal resection of the mandible with consideration of the possibility that the tumor was malignant.

To the best of our knowledge, PET findings of myofibroma have been reported in only one case in the literature. In that case, strong accumulation of FDG in a myofibroma of the mandible was documented [10]. The FDG-PET findings in the present case are in agreement with those of the previously reported case [10]. FDG accumulation could be caused partially by blood supply. For glucose to be taken up and used by tumor cells, an adequate blood supply is needed; therefore, FDG-PET is assessed by not only the glucose metabolism, but also the blood supply [12]. In our case, the strong FDG accumulation was assumed to be caused by activated glucose metabolism due to the high proliferative activity of tumor cells and rich blood supply to the tumor. High proliferative activity of tumor cells was proven macroscopically from the rapid growth of the tumor, and microscopically from the mitoses sometimes seen in the HE-stained sections. The rich blood supply to the tumor was evident from many blood vessels confirmed on the immunohistochemically stained sections, which showed CD34 positivity in vascular endothelial cells.

In the present case, gadolinium-enhanced T1-weighted MRI showed strong enhancement in the tumor. Strong enhancement on T1-weighted images by contrast material was also documented in some reported cases of myofibroma [13-15]. In the head and neck region, on the other hand, some tumors other than myofibroma (for example, hemangiomas, solitary fibrous tumors, carotid body tumors, and malignant tumors) also demonstrate strong contrast enhancement on T1-weighted images. However, hemangioma [16] and solitary fibrous tumor [17] could be discriminated through FDG-PET findings of weak accumulation of FDG within the lesions. Moreover, carotid body tumors can be clinically ruled out by the specific site of the tumor [18]. However, malignant tumors are difficult to differentiate from myofibroma on the basis of MRI and PET findings. To avoid unnecessarily aggressive surgery, the clinician should be aware that myofibroma could show false positive results in a PET study aiming for detection of malignant tumors. Although a preoperative specific diagnosis of myofibroma could not be established in our case, clinical findings in combination with MRI and PET could narrow down the differential diagnoses. Complete local excision is performed for the treatment of solitary myofibroma [6]. Recurrence is rare, and such cases are usually controlled with re-excision [1,3]. In the present case, the tumor was completely removed by marginal resection of the left mandible and we found no evidence of recurrence at the postoperative 34-month follow-up.

## Conclusions

In this report, we described an adult case of myofibroma that arose on the mandible and discussed the diagnostic value of preoperative MRI and PET findings.

## Consent

Written informed consent was obtained from the patient for publication of this case report and any accompanying images. A copy of the written consent is available for review by the Editor-in-Chief of this journal.

## Abbreviations

CT: Computed tomography; FDG: ^18^ F-fluorodeoxyglucose; HE: Hematoxylin and eosin; MRI: Magnetic resonance imaging; PET: Positron emission tomography.

## Competing interests

The authors declare that they have no competing interests.

## Authors’ contributions

YT and TS performed the operation. HF and TS were clinically responsible for the patient’s care. KM was responsible for the pathology. KK and KN reviewed the literature. YT and HY wrote the main manuscript. YH revised the manuscript for important intellectual content. All authors read and approved the final manuscript.
